# Supplementary material for: Comparative Analysis of Two Autophagy-Enhancing Small Molecules (AUTEN-67 and -99) in a Drosophila Model of Spinocerebellar Ataxia Type 1
Source: Int J Mol Sci. 2025 Oct 27;26(21):10443. doi: 10.3390/ijms262110443 (PMC12609819; doi:10.3390/ijms262110443)
Supplement: Supplementary file 1 [file ijms-26-10443-s001.zip › ijms-3919197-supplementary.pdf]

Supplementary Figure legends

Figure S1

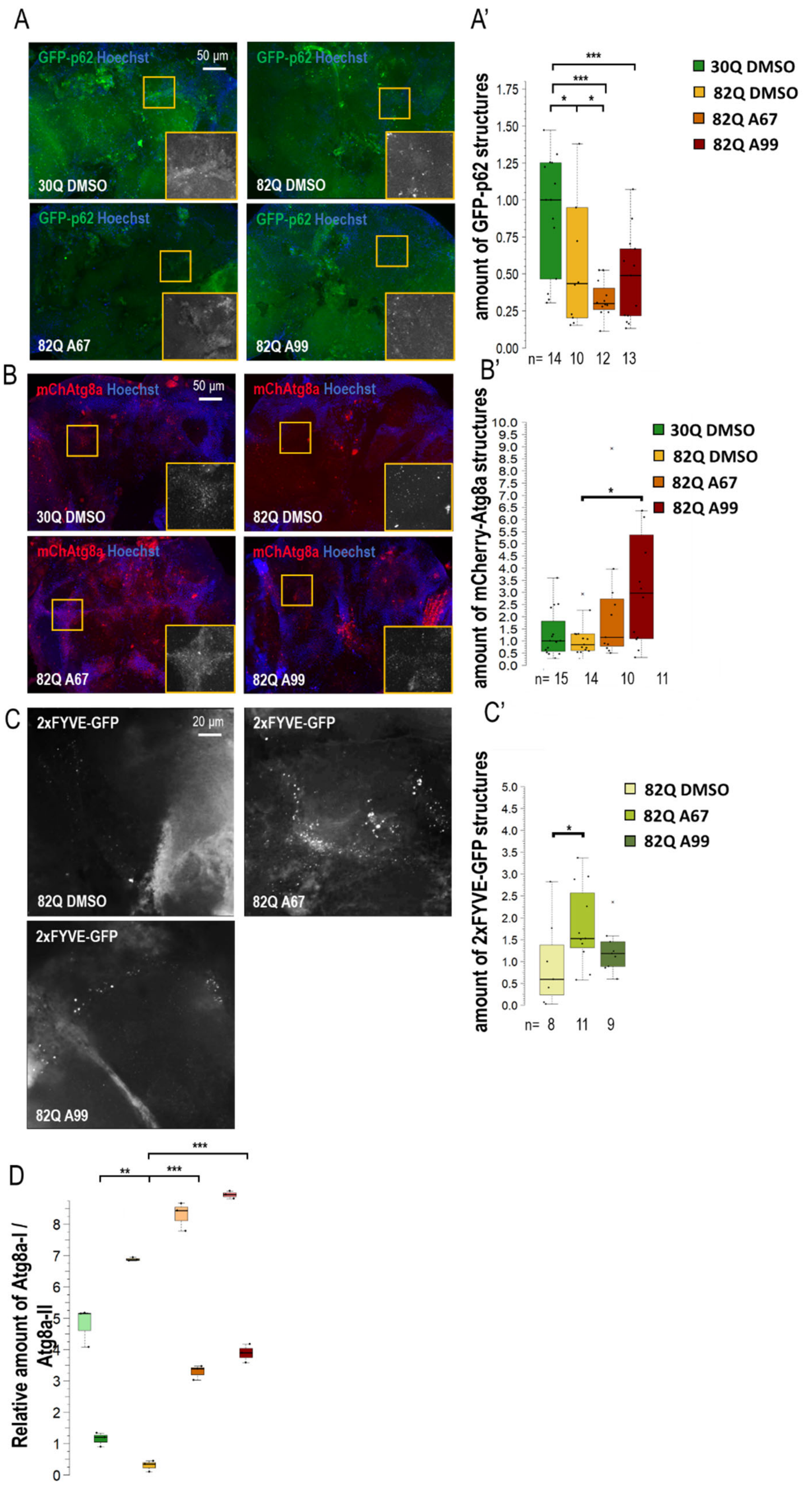

Figure S1. AUTEN-67 reduces GFP-p62-positive aggregates in SCA1 model. **(A)** GFP-p62/Ref(2)P reporter was used to quantify the level of autophagic degradation. 82Q ATXN1-expressing neurons were compared with 30Q ATXN1-expressing control neurons. Control was treated with DMSO (AUTEN molecules were dissolved in DMSO). Yellow squares indicate the enlarged areas. **(B-B')** mCherry-Atg8a (red) marker was used to measure the amount of autophagy compartments. We measured a significant increase in mCherry-Atg8a structure abundance in AUTEN-99 treated 82Q animals. **(C-C')** We also examined changes in 2xFYVE-GFP in the whole *Drosophila* brain. AUTEN-67 significantly increases the amount of FYVE-positive structures. **(D)** The diagram shows the Western blot evaluation of Figure1 C. On the box-plot diagrams, the black line indicates the median value, the box shows the most representative 50%, while filed black circles show the value of individual samples. The "n" indicates the number of samples used per treatment. Starts indicate P values where *: $p < 0.05$.

Figure S2

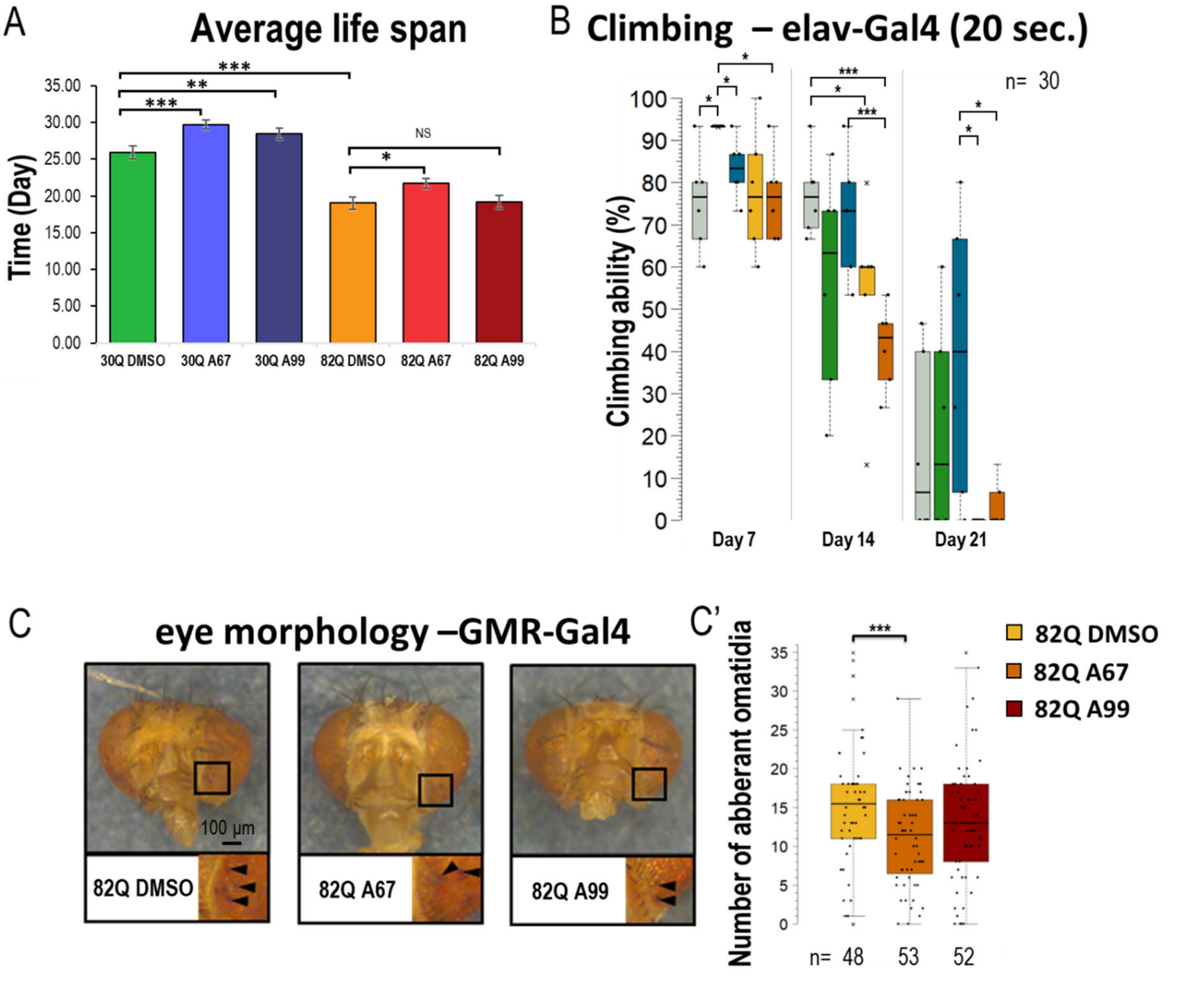

Figure S2. Both AUTENs have lifespan-prolonging effects in wild-type ATXN1 animals, whereas only AUTEN-67 is effective in ATXN1 mutants. **(A)** The mean lifespan values in the graphs are the Kaplen-Meier lifespan curves in Figure 2. Drug treatments were performed in animals expressing wild-type (30Q) and mutant (82Q) ATXN1 protein in the nervous system (*elav-Gal4*). AUTEN-67 (A67) and AUTEN-99 (A99) were dissolved in DMSO. Therefore, controls were also treated with DMSO in the same volume as the drugs. **(B)** Animals 30Q and 82Q expressing *elav-Gal4* were also tested for locomotion. The figure shows the results of a long distance (21.8 cm) measurement of the animals over 20 s. A67 significantly reduces the amount of abberant omatidia. **(C and C')** We treated the larvae on a medium containing AUTEN molecules (in nutrient), and after hatching, we counted the aberrant ommatidia. Only A67 has decreases the number of aberrant ommatidia. On the box-plot

diagrams, the black line indicates the median value, the box shows the most representative 50%, while filed black circles show the value of individual samples. The "n" indicates the number of samples used per treatment. Starts indicate P values where *: $p < 0.05$.

Figure S3

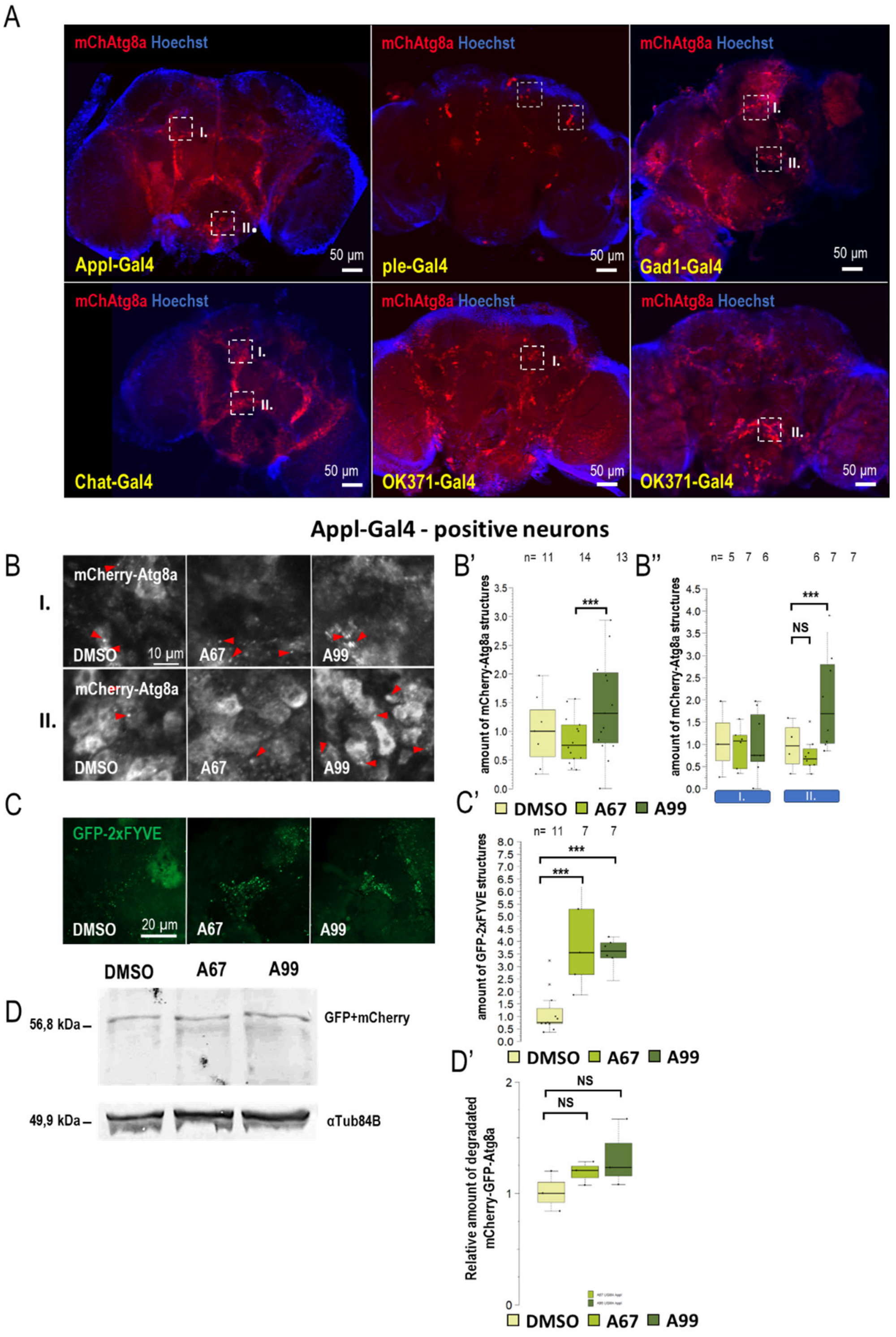

Figure S3. AUTEN-99 treatment increases the amount of autophagic structures to a greater extent than AUTEN-67 when tested in the whole brain (except for the eye lobes). (A) We examined quantitative changes in mCherry-Atg8a using five different expression patterns of nerve-specific Gal4: *Appl-Gal4* (whole brain except eye lobe), *ple-Gal4* (dopaminergic neurons), *Gad1-Gal4* (GABAergic neurons), *Chat-Gal4* (cholinergic neurons) and *OK371-Gal4* (glutaminergic neurons and motoneurons). The expression patterns of these *Gal4s* are shown in the figure. The areas indicated by the white dashed line show the measurement areas. **(B** and **B')** When examined with *Appl-Gal4*, the amount of mCherry-Atg8a structures increased significantly only in AUTEN-99 (A99) treated animals. **(B")** This change increased significantly in subpharyngeal neurons. **(C** and **C')** The GFP-2xFYVE marker can be used to monitor the amount of PI3P and infer the efficiency of vesicle nucleation. The GFP-2xFYVE marker was expressed with *Appl-Ga4* in the adult *Drosophila* brain. Both agents significantly increased the amount of GFP-2xFYVE structures. **(D** and **D')** Western blot labeling was used to determine the number of cleaved forms of GFP-mCherry-Atg8a in protein samples from the heads of DMSO- and AUTEN-treated animals. αTubulin labeling was used as an internal control. Both AUTEN treatments increased the amount of GFP-mCherry cleaved protein form, indicating activation of autophagy. On the box-plot diagrams, he black line indicates the median value, the box shows the most representative 50%, while filed black circles show the value of individual samples. The "n" indicates the number of samples used per treatment. Starts indicate P values where *: $p < 0.05$, ** : $p < 0.01$, *** : $p < 0.005$.

Figure S4

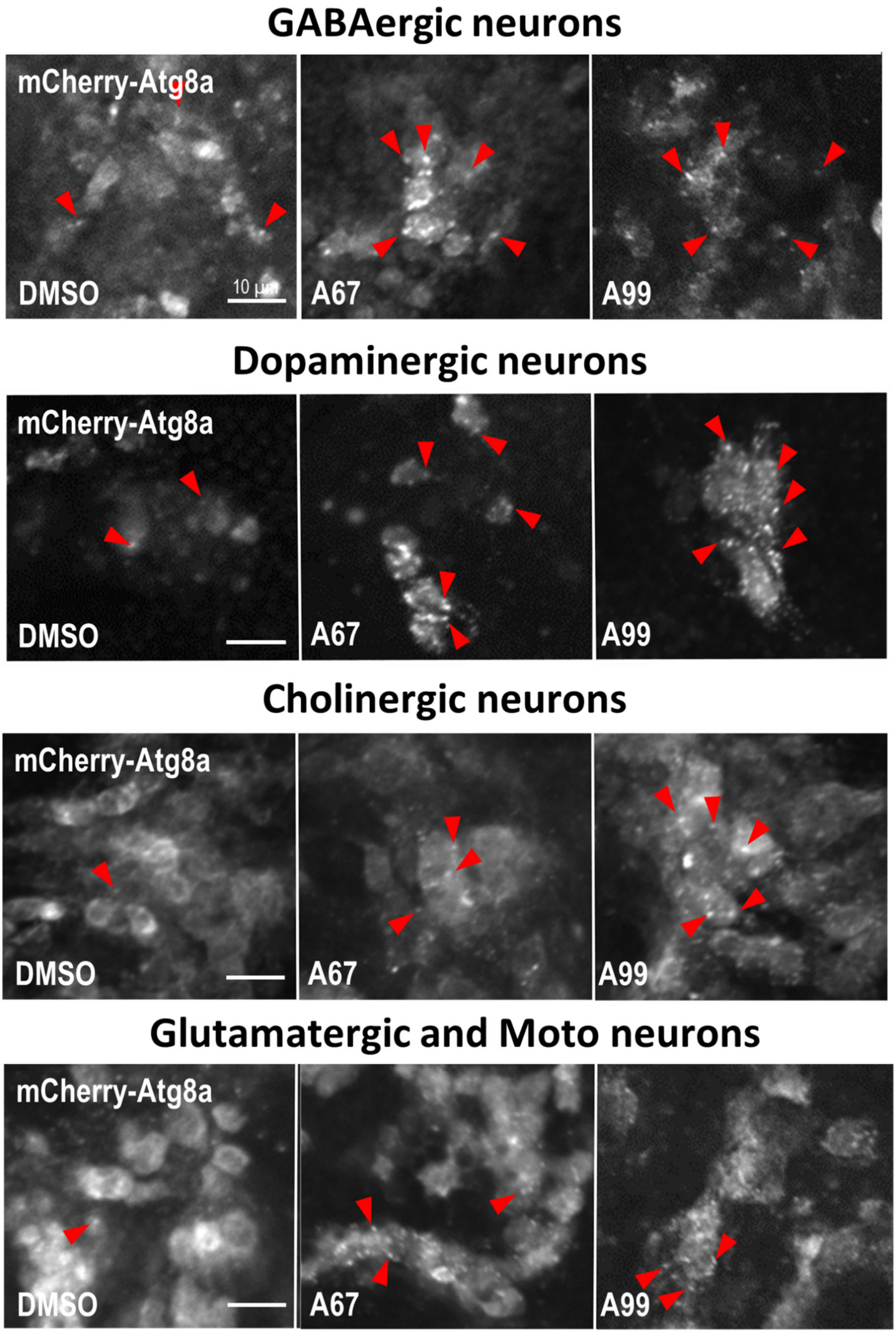

GABAergic neurons
mCherry-Atg8a
DMSO
10 μm
A67
A99
Dopaminergic neurons
mCherry-Atg8a
DMSO
A67
A99
Cholinergic neurons
mCherry-Atg8a
DMSO
A67
A99
Glutamatergic and Moto neurons
mCherry-Atg8a
DMSO
A67
A99

Figure S4. The mCherry-Atg8a positive structures in different neuron types after drug treatment (DMSO, A67, A99). The picture shows enlarged parts of Figures 3A and B and Figures 4A and B.

Figure S5

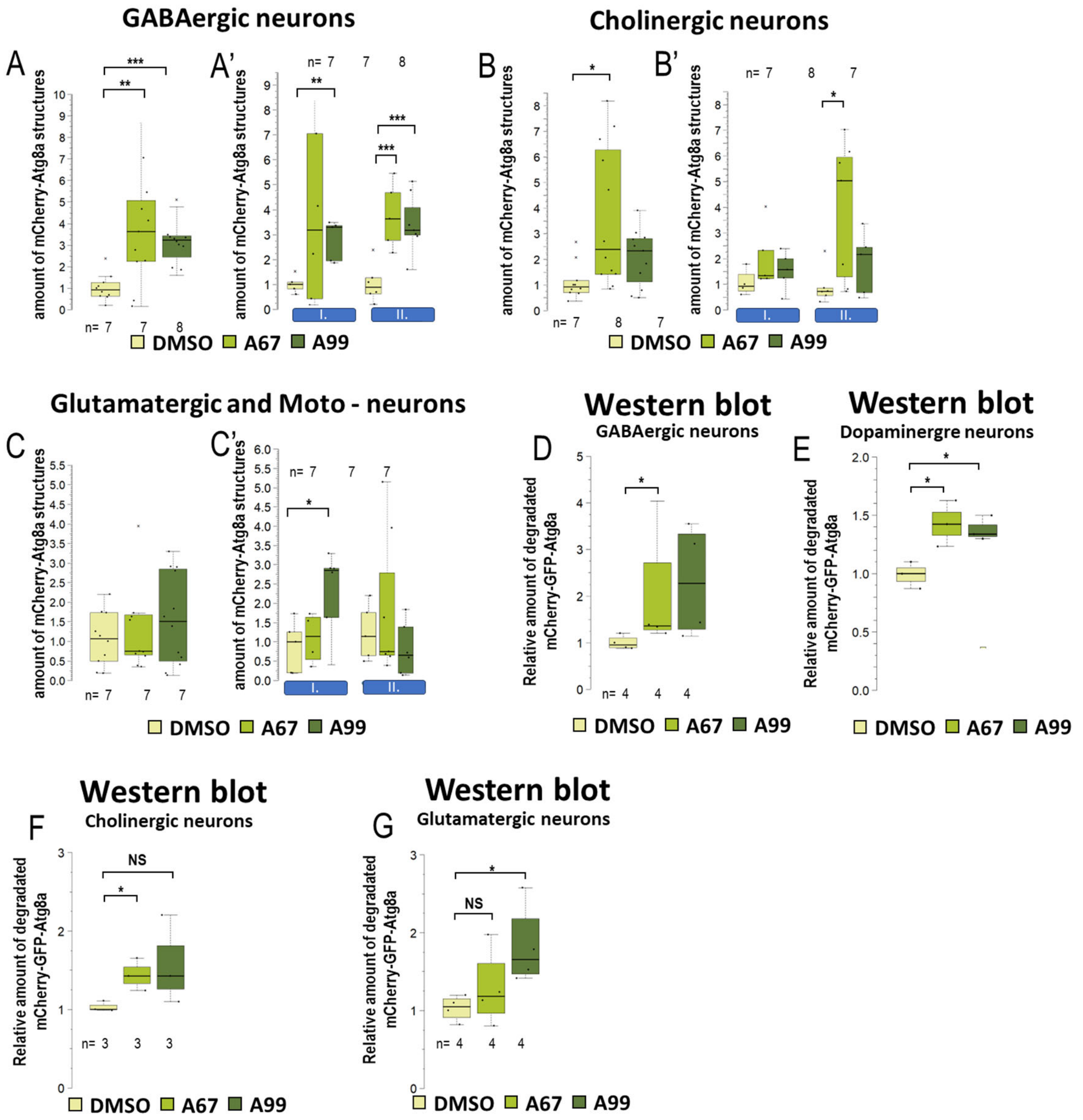

Figure S5. In GABAergic neurons, both drug candidates increase the number of mCherry-Atg8a structures, whereas in cholinergic neurons only AUTEN-67 (A67) while in glutaminergic and motor neurons AUTEN-99 (A99) cause significant changes. (A to **C')** Using different neuron-specific drivers, we investigated the amount of autophagic vesicles in 2 brain areas (I- mushroom body, II- subpharyngeal nerve). **(A** and **Ai)** Both A67 and A99 significantly increase the amount of autophagic structures in total **(A)** and measured separately **(Ai)** in the two areas. **(B** and **B')** A67 significantly increases the amount of mCherry-Atg8a in cholinergic neurons in the mushroom body area. **(C** and **Ci)** In glutamatergic and motor neutrons, only A99 increases the amount of autophagic structures. **(D** to **G)** To measure the autophagy flux, the degradation products of mCherry-GFP-Atg8a protein were analyzed by western blotting. The diagrams show the total amount of GFP-positive fluorescent proteins (free mCherry-GFP and GFP) cleaved during degradation. Figures for the diagrams are shown in Figures 3 and 4. On the box-plot diagrams, he black line indicates the median value, the box shows the most representative 50%, while filed black circles show the value of individual samples. The "n" indicates the number of samples used per treatment. Starts indicate P values where *: $p < 0.05$, ** : $p < 0.01$, *** : $p < 0.005$.

Figure S6

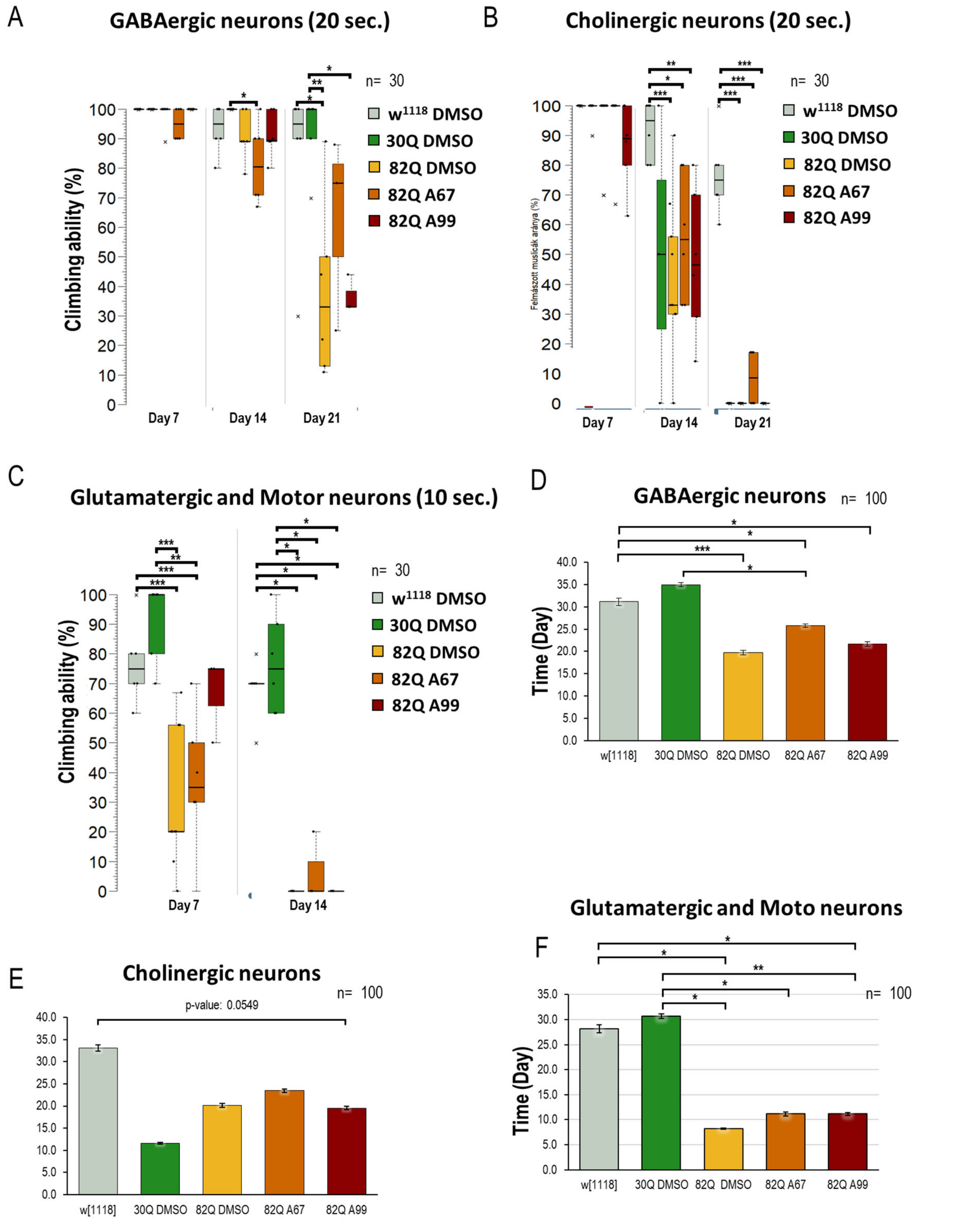

Figure S6. AUTEN-67 and -99 improve climbing ability in GABAergic, Glutaminergic, and motor neuron-specific ATXN1 mutants. (A and **B)** The effects of AUTEN-67 (A67) and AUTEN-99 (A99) on short-term (6 cm) locomotion were studied in animals maintained at 29°C for 7, 14 and 21 days. The 82Q animals were unable to climb 21.8 cm in 1 min (long-distance climbing), so we changed the measurement to short-distance, where we measured the animals' climbing for 10 and 20 s. In these animals, the ATXN1 (82Q) mutation was expressed in GABAergic **(A)** and cholinergic **(B)** neurons. **(C)** Specific expression of 82Q in glutaminergic and motor neurons resulted in a short shortened life span. Thus, we could only perform climbing assays in animals (kept at 29°C) for 7 and 14 days. The 82Q control animals (treated with DMSO only) did not survive 14 days. Both groups of 82Q

animals treated with AUTEN were still able to climb on day 14. The additional data for Figure 5 can be found in Figure S5. On the box-plot diagrams, he black line indicates the median value, the box shows the most representative 50%, while filed black circles show the value of individual samples. The "n" indicates the number of samples used per treatment. Starts indicate P values where *: $p < 0.05$, ** : $p < 0.01$, *** : $p < 0.005$.

Figure S7

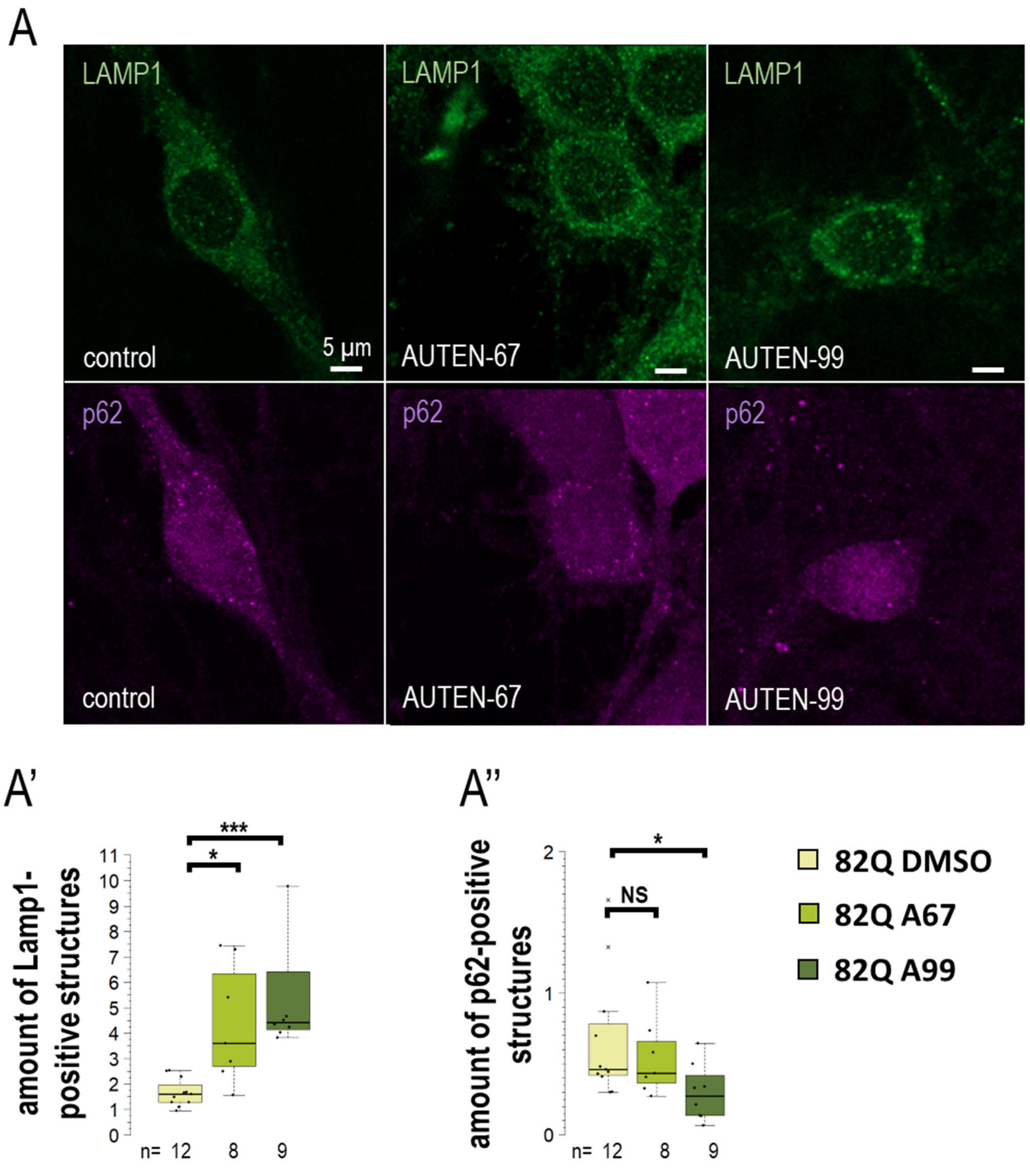

Figure S7. AUTNE-99 treatment increased the amount of Lamp1- and p62-positive structures in mouse hippocampal neurons. Based on their shapes, we can clearly distinguish different types of neurons from the glial cells. The smaller, rounder cells are GABAergic neurons in which we compared the amount of the lysosomal substrate Lamp1(green) and the amount of the autophagic substrate p62 (purple) after treatments (DMSO-control, A67, A99). On the box-plot diagrams, he black line indicates the median value, the box shows the most representative 50%, while filed black circles show the value of individual samples. Starts indicate P values where *: $p < 0.05$, ** : $p < 0.01$, *** : $p < 0.005$.
